# Opsonization of malaria-infected erythrocytes activates the inflammasome and enhances inflammatory cytokine secretion by human macrophages

**DOI:** 10.1186/1475-2875-11-343

**Published:** 2012-10-09

**Authors:** Jingling Zhou, Louise E Ludlow, Wina Hasang, Stephen J Rogerson, Anthony Jaworowski

**Affiliations:** 1 Centre for Virology, Burnet Institute, PO Box 2284, Melbourne, Victoria, 3001, Australia; 2 Department of Medicine, Monash University, Melbourne, Vic, 3004, Australia; 3 Department of Immunology, Monash University, Melbourne, Vic, 3004, Australia; 4 Department of Medicine (RMH), Centre for Medical Research, Royal Melbourne Hospital, University of Melbourne, Melbourne, Vic, 3050, Australia; 5 Victorian Infectious Diseases Service, Royal Melbourne Hospital, Parkville, Vic, 3050, Australia

**Keywords:** *Plasmodium falciparum*, Human, Monocyte-derived macrophages, Antibody, Fc gamma receptor, Phagocytosis, Pro-inflammatory cytokines

## Abstract

**Background:**

Antibody opsonization of *Plasmodium falciparum*-infected erythrocytes (IE) plays a crucial role in anti-malarial immunity by promoting clearance of blood-stage infection by monocytes and macrophages. The effects of phagocytosis of opsonized IE on macrophage pro-inflammatory cytokine responses are poorly understood.

**Methods:**

Phagocytic clearance, cytokine response and intracellular signalling were measured using IFN-γ-primed human monocyte-derived macrophages (MDM) incubated with opsonized and unopsonized trophozoite-stage CS2 IE, a chondroitin sulphate-binding malaria strain. Cytokine secretion was measured by bead array or ELISA, mRNA using quantitative PCR, and activation of NF-κB by Western blot and electrophoretic mobility shift assay. Data were analysed using the Mann–Whitney U test or the Wilcoxon signed rank test as appropriate.

**Results:**

Unopsonized CS2 IE were not phagocytosed whereas IE opsonized with pooled patient immune serum (PPS) were (Phagocytic index (PI)=18.4, [SE 0.38] n=3). Unopsonized and opsonized IE induced expression of TNF, IL-1β and IL-6 mRNA by MDM and activated NF-κB to a similar extent. Unopsonized IE induced secretion of IL-6 (median= 622 pg/ml [IQR=1,250-240], n=9) but no IL-1β or TNF, whereas PPS-opsonized IE induced secretion of IL-1β (18.6 pg/mL [34.2-14.4]) and TNF (113 pg/ml [421–17.0]) and increased IL-6 secretion (2,195 pg/ml [4,658-1,095]). Opsonized, but not unopsonized, CS2 IE activated caspase-1 cleavage and enzymatic activity in MDM showing that Fc receptor-mediated phagocytosis activates the inflammasome. MDM attached to IgG-coated surfaces however secreted IL-1β in response to unopsonized IE, suggesting that internalization of IE is not absolutely required to activate the inflammasome and stimulate IL-1β secretion.

**Conclusions:**

It is concluded that IL-6 secretion from MDM in response to CS2 IE does not require phagocytosis, whereas secretion of TNF and IL-1β is dependent on Fcγ receptor-mediated phagocytosis; for IL-1β, this occurs by activation of the inflammasome. The data presented in this paper show that generating antibody responses to blood-stage malaria parasites is potentially beneficial both in reducing parasitaemia via Fcγ receptor-dependent macrophage phagocytosis and in generating a robust pro-inflammatory response.

## Background

There are an estimated 243 million clinical cases of malaria each year, resulting in almost 800,000 deaths (World Health Organization, World Malaria Report 2010). The burden of disease falls mainly on children under five years old and women in their first and second pregnancies (*ibid*, [1]). The majority of deaths in children, and morbidity associated with infection in pregnancy, are due to infection by *Plasmodium falciparum*. The production of antibodies to the blood stages of malaria parasites represents an important component of anti-malarial immunity. This is most clearly shown by the ability of passively transferred gamma globulin to clear blood-stage infection and alleviate clinical illness [2,3]. The mechanism of protection afforded by gamma globulins purified from hyperimmune donors is assumed to have occurred by transfer of malaria specific antibodies, however it cannot be ruled out that alleviation of disease symptoms may also be contributed by the immunomodulatory properties of intravenously administered gamma globulins [4]. The role of antibodies in protection from malaria is also shown by the protection afforded to newborn infants from maternal antibodies (although see [5]) and is suggested by an association of the titre of antibodies to malaria antigens with decreased risk of disease [6]. Protective antibodies are directed against merozoite proteins and variant surface antigens expressed on infected erythrocytes (IE) [7,8].

The mechanism(s) by which antibodies confer protection needs to be defined in order to inform vaccine development. Mechanisms that have been proposed include inhibition of parasite growth [9-11], neutralization of surface proteins involved in merozoite entry into red blood cells [12-14], inhibition of IE sequestration [15], and promotion of parasite killing [16] or phagocytosis via Fcγ receptor-dependent mechanisms [16,17](reviewed in [18]). Antibody responses associated with protection in both children and pregnant women have been shown to be mainly comprised of cytophilic IgG antibodies of the subclasses IgG1 and IgG3 [19-26], which suggests a role for Fcγ receptors in protection. The role of Fcγ receptors in malaria immunity is supported by observations of a link between Fcγ receptor polymorphisms and outcomes of infection [27-30].

Erythrocytes infected with trophozoite-stage parasites are cleared in the spleen, liver and placenta by monocytes and monocyte-derived tissue macrophages (MDM).Antibodies to trophozoite-stage IE surface proteins opsonize IE and promote their removal by erythrophagocytosis. Engagement of Fcγ receptors on myeloid cells increases the efficiency of ingestion by phagocytosis and stimulates cytokine production, but may also alter the programme of inflammatory gene expression by macrophages in comparison to that induced by stimulation of innate immune receptors [31,32]. Pro-inflammatory cytokines are thought to have a role in limiting malaria parasitaemia in part via activation of the innate immune mechanisms of monocytes and macrophages. They may also play a role in immunopathogenesis, as originally postulated by Clark and co-workers from their studies on mouse models of malaria [33]. This dual potential is illustrated by mouse models of malaria infection in which the effect of loss of pro-inflammatory cytokine production in IRAK4−/− mice, whose monocytes have defective cytokine production due to loss of signalling from multiple toll-like receptors, has either a beneficial or deleterious effect on outcomes depending upon the susceptibility of the mouse strain [34]. It is, therefore, crucially important to understand how opsonization by immune serum affects pro-inflammatory cytokine production in response to malaria antigens.

As part of studies to investigate how opsonization of IE affects cytokine production by MDM, and how HIV infection of MDM may alter these responses [35], the influence of opsonization with immune serum on the production of the pro-inflammatory cytokines IL-1β, TNF and IL-6, by human MDM exposed to trophozoite-stage *P. falciparum* IE was investigated. To avoid complications of non-opsonic phagocytosis via CD36, the CS2 parasite strain [36], a model for chondroitin sulphate A (CSA) binding maternal malaria parasites which does not bind to CD36, was studied. It is shown that mRNA encoding IL-1β, TNF and IL-6 is induced by unopsonized IE in the absence of phagocytosis, and that opsonization with IgG enhances phagocytosis and IL-6 protein secretion, and enhances IL-1β secretion via activation of the inflammasome. These data show that pro-inflammatory cytokine gene expression is activated via surface-expressed innate immune receptors and requires additional signals, which may be derived from Fcγ receptor signalling pathways or internal pattern recognition receptors, to promote robust pro-inflammatory cytokine secretion.

## Methods

### Isolation of monocytes and culture of monocyte-derived tissue macrophages

Human peripheral blood mononuclear cells (PBMC) were obtained from Buffy Coats separated from volunteer blood donations (Australian Red Cross Blood Service, Southbank, Victoria, Australia) using Ficoll-Paque™ density gradient centrifugation. Monocytes were isolated from PBMC by countercurrent elutriation using a Beckman J-6M/E centrifuge equipped with a JE 5.0 rotor and tested for purity as described previously [37]. MDM were prepared by culturing freshly isolated monocytes adhered to plastic in Iscove’s modified Dulbecco’s medium (Invitrogen) containing 10% heat-inactivated human serum (Red Cross Blood Service, Sydney, Australia) supplemented with 2 mM glutamine, 100 U/mL penicillin G and 100 μg/mL streptomycin sulphate (IH10 medium). MDM were cultured as described [35], and where indicated they were activated for 48 hr with 100 ng/ml human IFN-γ (R&D Systems).

### Preparation and opsonization of CS2 IE

The CSA binding *P. falciparum* strain CS2 [36] was maintained in unexpired human group O+ erythrocytes (Australian Red Cross Blood Service) in RPMI-HEPES supplemented with 0.5% Albumax II (GIBCO) and 25 mM NaHCO_3_ and tested for CSA adhesion and Mycoplasma contamination as described [17,35]. Mature trophozoite-stage IE were purified from discontinuous Percoll gradients as described [17,35]. IE collected from the 60% layer (92-95% purity) were washed three times and re-suspended in PBS at a density of 1x10^8^ per ml then opsonized for 30 min at room temperature with 9% pooled patient immune serum (PPS) from Malawian HIV-uninfected pregnant women with malaria, which demonstrated high levels of antibody to CS2 IE [17], or left unopsonized. In some experiments, IE were opsonised as above with 10% non-immune human serum prepared from pooled serum from healthy Australian donors (provided by the Australian Red Cross Blood Service). IE were washed and re-suspended in PBS at 1x10^8^/ml and used immediately.

### Measurement of phagocytosis

IE were added to MDM cultured in 96-well plates at a target to cell ratio of 20:1 unless otherwise indicated, then incubated for 1 hr. The extent of phagocytosis was determined by measuring internalized haemoglobin using a colorimetric assay as described [17][35,38]. The haemoglobin content was converted to equivalents of erythrocytes ingested by reference to a standard curve of known amounts of IE from the same preparation, and phagocytosis expressed as a phagocytic index representing erythrocytes ingested per 100 MDM.

### Measurement of cytokine gene expression and protein secretion

MDM were cultured in 96-well plates and exposed in triplicate to IE under varying conditions of opsonization for 24 hr. Media from triplicate wells were collected, pooled, then analysed for cytokines using a cytokine bead array (BD Biosciences, Human Inflammatory Cytokine Kit). In some experiments, culture medium was analysed for IL-6 secretion using an ELISA assay (Mabtech AB). To measure mRNA expression, MDM were cultured in 24-well plates and exposed to IE for various times then lysed using lysis buffer A (0.1 M Tris HCl, pH 7.5 containing 1% lithium dodecyl sulphate, 0.5 M LiCl, 10 mM EDTA, 5 mM DTT) to extract total cellular RNA. Cellular mRNA was isolated from extracts using oligo(dT) magnetic beads (GenoPrep^TM^, GenoVision), and cDNA was prepared using a Transcriptor First Strand cDNA Synthesis Kit (Roche) followed by amplification of cytokine cDNAs by quantitative real-time PCR in Brilliant® II SYBR® Green qPCR Master Mix (Stratagene) using primer pairs for TNF, IL-1β and IL-6 and amplifications as previously described [35,39].

### Western blot detecting nuclear localization of NF-κB subunits

MDM (1x10^6^ per 6 cm dish) were primed with IFN-γ for 48 hr and treated with media alone, IE or IE opsonized with PPS for 24 hr, or with 1 ng/ml LPS for 2 hr followed by preparation of nuclear and cytoplasmic extracts using NE-PER® Nuclear and Cytoplasmic Extraction Reagents and Halt™ Protease and Phosphatase Inhibitor Cocktail, EDTA-free, according to the manufacturer’s protocol (Pierce Biotechnology). Protein concentration was determined using the Lowry method (BioRad) and 100 μg protein was boiled in protein loading buffer and separated by SDS-PAGE for immunoblot analysis. Protein was transferred onto nitrocellulose, and probed with antibodies as follows: rabbit anti-NF-κB p105/p50 (#3035, 1:1000) (Cell Signalling Technologies), rabbit anti-NF-κB p65 (C20, 1:1000) (Santa Cruz Biotechnology), rabbit anti-TATA binding protein TBP (ab63766, 1:1000) (AbCam) and mouse anti-GAPDH (6C5, 1:2500) (Santa Cruz Biotechnology). Primary antibody incubations were performed overnight at 4°C. Secondary antibodies used were HRP-conjugated donkey anti-rabbit and sheep anti-mouse IgG (GE Healthcare, Amersham) and detection was performed with enhanced chemiluminescence reagent (GE Healthcare, Amersham).

### Electrophoretic mobility shift assay (EMSA)

Single-stranded DNA oligonucleotides were generated containing NF-κB consensus sequence: Forward: 5’ AGTTGAGGGGACTTTCCCAGGC 3’ and Reverse: 5’ GCCTGGGAAAGTCCCCTCAACT 3’. In addition, NF-κB mutant oligonucleotides were synthesised: Forward: 5’ AGTTGAGGCGACTTTCCCAGGC 3’ and Reverse: 5’ GCCTGGGAAAGTCGCCTCAACT 3’ (GeneWorks). NF-κB consensus sequence oligonucleotides were labelled using the Biotin 3’-End DNA Labelling Kit (Pierce Biotechnology) and annealed. Unlabelled and mutant NF-κB oligonucleotides were annealed for use in competition experiments. NF-κB binding activity was determined using the LightShift Chemiluminescent EMSA Kit (Pierce Biotechnology). Nuclear protein extract (5 μg protein) was incubated for 40 min in binding buffer containing 200 ng poly dI:dC, 1% NP-40 and 50% glycerol with labelled probe. For competition assays, 100-fold molar excess of unlabelled NF-κB probe or its mutant probe was added 20 min prior to labelled probe as described [40]. For supershift assays, 2 μg of rabbit polyclonal antibody against NF-κB p65 (H-286) or p50 (H-119, Santa Cruz Biotechnology) or control antibody were incubated with nuclear extracts 20 min prior to adding labelled probe [40]. Complexes were resolved using 4-20% Tris-Borate EDTA (TBE) native polyacrylamide gels (Lonza) in 0.5x TBE running buffer at 4°C and transferred to Biodyne® B nylon membrane (Pierce Biotechnology). Detection was achieved using the Chemiluminescent Nucleic Acid Detection Module (Pierce Biotechnology).

### Fcγ receptor cross-linking experiments

Wells of 96-well plates were coated with 1 mg/ml human IgG (kind gift of Prof M Hogarth) overnight at 4°C then washed twice with calcium and magnesium free PBS. MDM grown for 7 days under non-adherent conditions in Teflon jars (Minnetonka), and primed with IFN-γ for 48 h, were added at 50,000 per well and adhered for 30 min. Cells were washed twice with PBS then exposed to 0.5-2.0 x10^6^ unopsonized CS2 IE for 24 hr (10–40:1 ratio of target to MDM). Culture medium was collected, and triplicate wells were pooled for measurement of cytokine secretion as described. Parallel wells were also seeded with 50,000 MDM in triplicate to measure phagocytosis of IE and opsonized IE.

### Analysis of caspase 1 activity

IFN-γ primed MDM cultured in 6 cm dishes were exposed for 4 hr to 6x10^7^ CS2 IE opsonized with sub-agglutinating concentrations (1:600) of rabbit anti-human erythrocyte antibody (MP Biomedicals) or to an equal number of unopsonized IE. Unbound IE were removed by washing once with ice-cold PBS, then bound but uningested IE were removed by lysis for 3 min with 0.2% NaCl followed by washing in ice-cold PBS. MDM were lyzed with 100 μL RIPA buffer (25 mM Tris–HCl (pH 7.5), 0.14 M NaCl, 1 mM EDTA, 0.1% SDS) supplemented with protease inhibitors (Roche, Complete protease inhibitor cocktail) and phosphatase inhibitors (50 mM NaF, 1 mM sodium orthovanadate, 40 mM β-glycerophosphate) for 15 min at 4°C. Lysates were clarified by centrifugation (20,000 *xg,*/10 min, 4°C) and 50 μg of protein was analysed by immunoblotting for caspase-1 activation (anti caspase-1 p10, sc-515, 1:400, Santa Cruz Biotechnology) using 15% acrylamide gels. Gels were re-probed with mouse anti-GAPDH (6C5, 1:2500, Santa Cruz Biotechnology) as a loading control. To measure the effect of caspase inhibition on cytokine secretion, MDM cultured in 96-well plates were pre-incubated for 2 hr with 10 μM benzyloxycarbonyl Val-Ala-Asp (zVAD)–FMK before stimulation with opsonized or unopsonized CS2 IE, and cytokine secretion was measured as described after 24 hr.

To measure caspase enzymatic activity, IFN-γ-primed MDM cultured in 6 cm dishes were exposed to opsonized or unopsonized CS2 IE at a ratio of 20 IE per cell for 0–4 hr. Extracts were prepared and assayed using a fluorogenic substrate according to manufacturer’s protocol (Caspase-1 fluorometric assay, R&D systems, BF12100).

### Statistical analysis

Statistical significance between groups was calculated using the Mann–Whitney non-parametric U test or, for paired comparisons, the Wilcoxon signed rank test. All statistical analyses were carried out using Prism 5.0 software (GraphPad Software). Significance was assumed when probability value was <0.05 in all cases.

### Ethics

Sera used to produce the positive pool were collected as part of studies approved by the College of Medicine Research Ethics Committee, Blantyre, Malawi and the Melbourne Health Human Research Ethics Committee, Melbourne.

## Results

### Phagocytosis of trophozoite-stage CS2 IE

**Figure 1:**
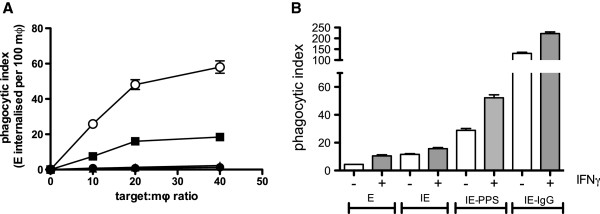
**Opsonized but not unopsonized CS2 IE are ingested by IFN-γ primed and unprimed MDM.** (**A**) MDM were cultured for seven days in 96-well plates at 50,000 per well then exposed to human erythrocytes (●), human erythrocytes opsonized with rabbit anti-erythrocyte antibody (○), purified CS2 IE (▲) or purified CS2 IE opsonized with immune serum (■) at the indicated target to cell ratios. After 2 hr, the number of ingested erythrocytes was determined using phagocytosis assay as described in Methods and expressed as a phagocytic index (ingested erythrocytes per 100 MDM). Values represent mean ± SEM of triplicate determinations from a single donor monocyte preparation. (**B**) MDM were cultured as in (**A**) but incubated with (+) or without (−) 100 ng/ml IFN-γ between day 5 and day 7. MDM were then exposed to targets (20:1 target to cell ratio) for 1 hr and phagocytic indices determined. E: human erythrocytes, IE: CS2 trophozoites, IE-PPS: CS2 trophozoites opsonized with PPS, IE-IgG: CS2 trophozoites opsonized with rabbit anti-human erythrocyte IgG. Data represent mean ± SEM of quadruplicate determinations from a single donor monocyte preparation. Experiment is representative of two independent experiments.

To characterize phagocytic uptake of CS2 IE by MDM, seven-day adherent MDM cultures were exposed to uninfected erythrocytes (E) and to purified IE opsonized using various conditions and at varying target-to-effector ratios, and phagocytic indices were measured. Freshly isolated human erythrocytes were not ingested by MDM but were efficiently ingested when opsonized with rabbit anti-human erythrocyte antibody, which served as the positive control. IE were not ingested by MDM unless opsonized with PPS. Phagocytosis of IgG-opsonized targets reached a plateau at a target to macrophage ratio of 20:1 (Figure 1A) and this ratio was used in all subsequent experiments unless otherwise indicated. Priming of MDM with IFN-γ increased phagocytosis of antibody-opsonized CS2 IE without inducing phagocytosis of unopsonized CS2 IE (Figure 1B). Subsequent experiments were conducted using MDM primed with 100 ng/ml IFN-γ for 48 hr. In separate experiments it was shown that IE opsonised with non-immune serum were not phagocytosed at a greater rate compared to unopsonised IE (relative phagocytic index non-immune serum: unopsonised = 0.94, sd = 0.16, n=3).

### IL-1β, TNF and IL-6 mRNA are induced by unopsonized and opsonized CS2 IE

**Figure 2:**
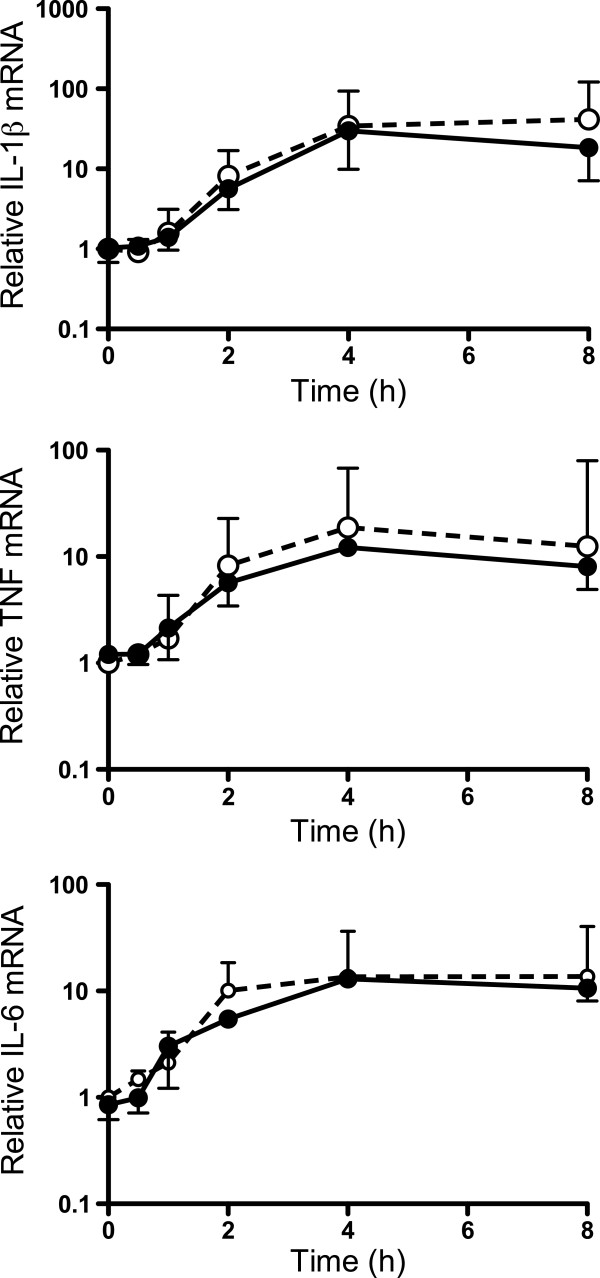
**Unopsonized and opsonized CS2 IE induce comparable pro-inflammatory cytokine mRNA expression.** MDM cultured in 24-well plates were primed with IFN-γ for 48 hr, and then exposed to purified CS2 IE opsonized with PPS(●) or without opsonization (○) at an IE to MDM ratio of 20:1. RNA extracts were prepared after the indicated times and analysed by qPCR for cytokine and GAPDH mRNA content as described in Methods. Cytokine mRNA was quantified using the comparative threshold method and expressed relative to levels at t=0. Values represent the median [± IQR] for six experiments using MDM prepared from separate donor monocytes. No significant differences between cells exposed to opsonized or unopsonized IE were observed at any time point (p<0.05, Mann Whitney U test comparing IE with IE-IgG at each time point).

**Figure 3:**
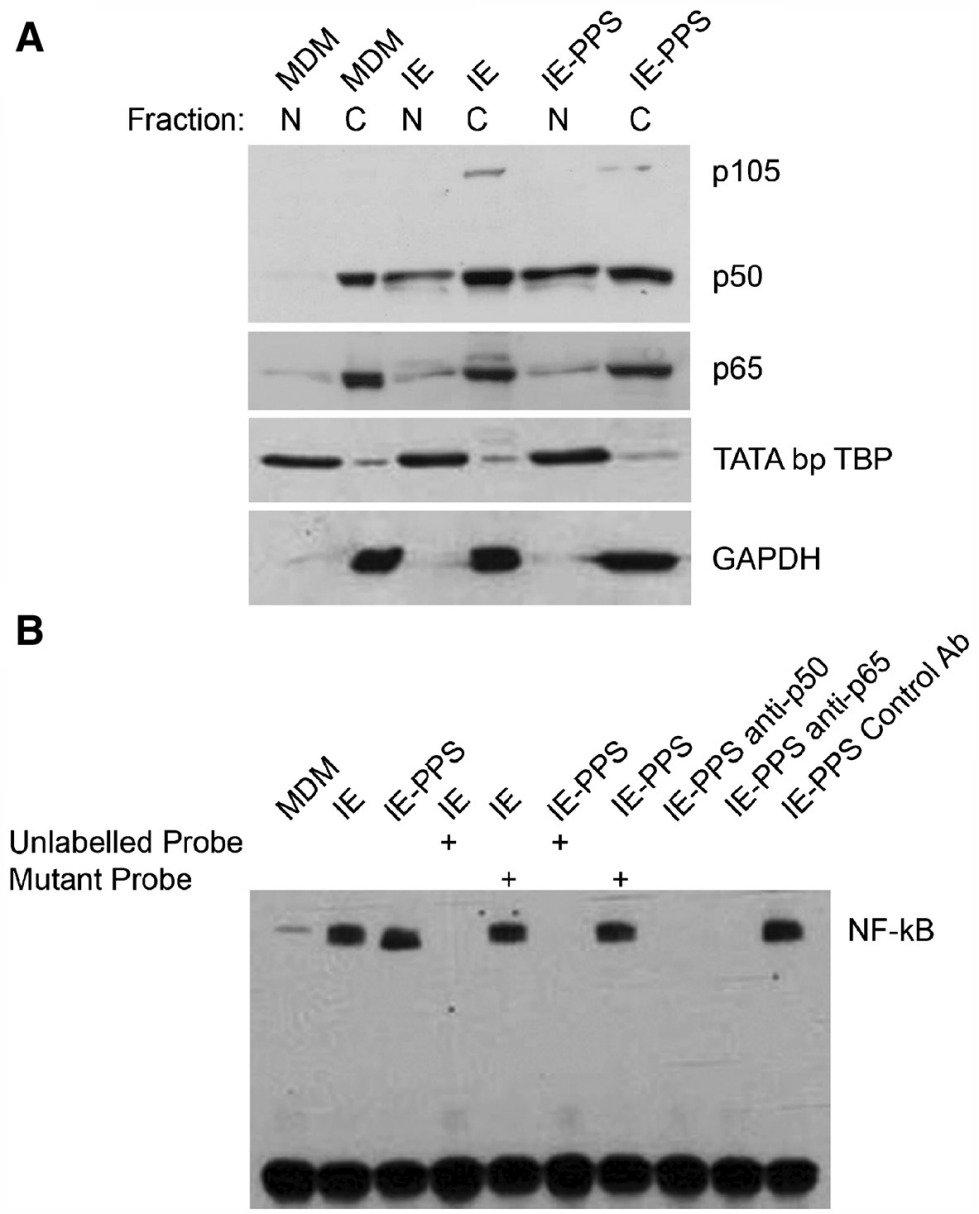
**Exposure of MDM to opsonized and unopsonized CS2 IE activates NF-κB to similar extents.** MDM (1x10^6^) were primed with IFN-γ for 48 hr, treated with media alone (MDM), 2x10^7^ unopsonized IE or IE opsonized with pooled immune patient serum (IE-PPS) for 24 hr followed by preparation of nuclear and cytoplasmic extracts. (**A**) Extracts were analysed by immunoblotting using antibodies detecting the p50 and p65 NF-κB subunits. Blots were re-probed using antibodies to GAPDH and TATA-binding protein (TBP) to assess the purity of cytoplasmic (**C**) and nuclear (N) fractions respectively. (**B**) IFN-γ-primed MDM were incubated with IE-PPS or IE or incubated in medium alone (MDM) for 24 hr, and nuclear extracts prepared as in (**A**). Binding of nuclear proteins to an NF-κB specific oligonucleotide was analysed using EMSA as described in Methods. Saturable binding was indicated by competition using 100-fold excess of unlabelled probe (lanes 4,6), specificity of binding by lack of competition using a mutant oligonucleotide probe (lanes 5,7) and the presence of p50 and p65 in DNA binding complexes confirmed by incubation with anti p50 and p65 or control monoclonal antibodies (lanes 8–10). The positions of complexes containing p50/p65 heterodimer are labelled NF-κB.

To determine whether ingestion of CS2 IE via phagocytosis was required to elicit a pro-inflammatory response, MDM were exposed to unopsonized and opsonized IE for various times, and mRNA encoding IL-6, TNF and IL-1β was measured. All mRNA species were induced after 30 min, and reached a peak at 4 hr (Figure 2). Significantly, the levels and kinetics of induction of mRNA for these cytokines were similar when MDM were exposed to unopsonized or opsonized IE, suggesting that ingestion was not required for a robust transcriptional response. Pro-inflammatory cytokine transcription in response to innate immune stimuli is regulated by NF-κB [41]. The ability of IE or opsonized IE to activate the NF-κB pathway was therefore determined. Activation was initially assessed by p50 (NF-κB1) and p65 (RelA) subunit translocation to the nucleus. Nuclear protein fractionation was validated using antibodies against the nuclear protein TATA binding protein TBP and the cytoplasmic protein GAPDH. Resting MDM contained low levels of p50 or p65 immunoreactivity in the nucleus (Figure 3A). Exposure to unopsonized CS2 IE resulted in accumulation of both p65 and p50 in nuclei, which was not enhanced when MDM were exposed to opsonized IE. The effect of IE or opsonized IE on NF-κB pathway activation was also assessed by electrophoretic mobility shift assay (EMSA) using an NF-κB consensus biotin-labelled oligonucleotide. Using the EMSA technique very low levels of specific NF-κB complexes were detected in nuclei of resting MDM, but these complexes were readily detected when MDM were exposed to CS2 IE and the levels of these complexes were not further enhanced upon exposure to opsonized CS2 IE (Figure 3B). Binding of the biotin-labelled NF-κB probe to nuclear extracts prepared from MDM stimulated with IE and opsonized IE was diminished following addition of 100-fold molar excess of unlabelled NF-κB probe. This competition experiment indicated specificity of the protein complex for the NF-κB consensus oligonucleotide. The mutant NF-κB probe did not compete with the biotin-labelled NF-κB probe indicating specificity. Furthermore, addition of anti-p50 and anti-p65 antibodies significantly diminished the NF-κB complex confirming the presence of these proteins in the oligonucleotide binding complex. Taken together, these data suggest that recognition of unopsonized trophozoite-stage CS2 IE by MDM is sufficient to stimulate NF-κB signalling and pro-inflammatory cytokine mRNA expression in the absence of phagocytosis.

### Pro-inflammatory cytokine secretion in response to IE

**Figure 4:**
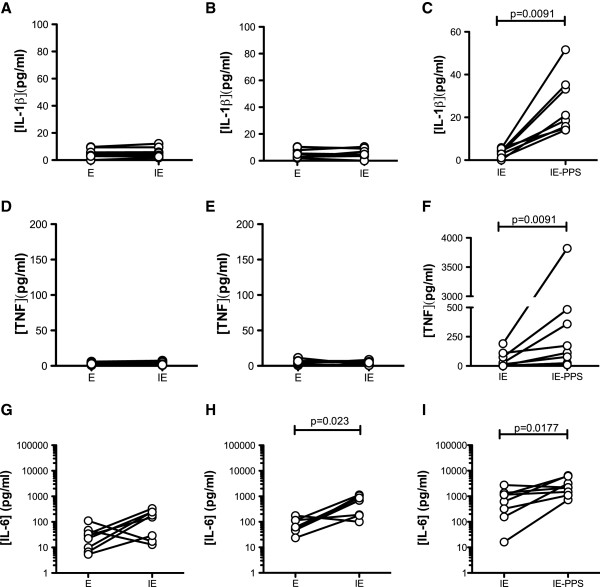
**Differential Secretion of IL-1β, TNF and IL-6 in response to opsonized *vs *unopsonized CS2 IE.** MDM from eight independent donor monocytes were cultured for seven days without 48 hr priming with 100 ng/ml IFN-γ (panels **A**, **D**, **G**) or with IFN-γ priming (panels **B**, **E**, **H**). MDM were exposed to human erythrocytes (E), CS2 IE or CS2 IE opsonized with PPS (IE-PPS) for 24 hr after which time the culture medium was analysed for IL-1β, TNF and IL-6 concentration as described in Methods. IFNγ-primed MDM from nine additional donors were exposed to CS2 IE or CS2 IE opsonized with PPS (IE-PPS) (panels **C**, **F**, **I**) for 24 hr and analysed as above. IL-6 concentrations are plotted on a log scale, whereas TNF and IL-1β are presented on linear scale. Differences were assessed for significance using Wilcoxon matched-pairs signed rank test.

To determine the effect of IFNγ priming and antibody opsonization on TNF, IL-1β and IL-6 secretion, MDM were incubated with human erythrocytes, IE or PPS-opsonized IE for 24 hr, and the concentrations of cytokines in culture medium were quantified. Unprimed MDM exposed to human erythrocytes as a negative control secreted no IL-1β and this was not increased following incubation with CS2 IE (median [IQR] = 3.98 [2.92-8.47] cf 5.49 [2.82-8.56] pg/ml, background values ~5 pg/ml in this assay, Figure 4A). Similar results were obtained using MDM primed with IFN-γ with the same targets (median [IQR] = 5.17 [2.80-9.61] cf 5.80 [3.81-9.23] pg/ml, p = 0.94, Figure 4B). Similarly, neither unprimed nor IFN-γ-primed MDM secreted TNF in response to IE (median [IQR] = 3.34 [2.04-4.82] cf 3.60 [1.63-6.51] pg/ml, p=0.30 for unprimed and median [IQR] = 6.11[3.67] cf 5.30 [2.70-7.01] pg/ml, p=0.81 for primed MDM, Figure 4D and E respectively). MDM exposed to human erythrocytes secreted low levels of IL-6 (26.0 [7.52-44.0] pg/ml) with a trend to increased secretion following incubation with unopsonized IE (169 [20.2-246] pg/ml, p = 0.078, Figure 4G). When IFN-γ-primed MDM were exposed to unopsonized IE there was a significant increase in IL-6 secretion compared to the erythrocyte control (692 [182–996] pg/ml cf 57.9 [46.6-115] pg/ml, p = 0.023, Figure 4H). In separate experiments, the impact of antibody opsonization of IE on cytokine release from IFNγ primed MDM was determined. Incubation of IFN-γ-primed MDM with PPS-opsonized IE resulted in secretion of IL-1β (median [IQR] = 18.6 [34.2-14.4] *cf* 3.48 [4.88-1.07] pg/ml p=0.0091 compared to IE, Figure 4C). Incubation of IFN-γ-primed MDM with opsonized IE resulted in TNF secretion (median [IQR] = 113 [421–17.0] pg/ml, compared with 14.0 [92.8-1.21] pg/ml for unopsonized IE, p=0.0091, Figure 4F). The amount of IL-6 secreted was further increased on exposure to PPS-opsonized IE (2195 [4658–1095] pg/ml cf 622 pg/ml [1250–240], p = 0.0177, Figure 4I). Taken together these data show that exposure to unopsonized IE elicited secretion of IL-6 but not TNF or IL-1β, and exposure to IE opsonized with immune serum increased secretion of all cytokines. These levels compare with a median of 33.5 [41.6-20.8] pg/ml IL-1β, 9,120 [10,600-8,130] pg/ml IL-6 and 1,960 [3,230-1,160] pg/ml TNF produced in response to the positive control 1 ng/ml LPS (data not shown). As an additional control it was shown in separate experiments that the levels of TNF, IL-1β and IL-6 secreted in response to IE opsonised with non-immune sera were similar to those secreted in response to unopsonised IE (TNF: 518.8 [1184–490.0] pg/ml cf 648.6[1195–487.9] pg/ml; IL-1b: 50.70 [159.0-39.77] pg/ml cf 58.89 [170.4-43.25] pg/ml; IL-6: 5895 [7525–4899] pg/ml cf 6583[7381–4690] pg/ml, n=3). Secretion of IL-10 and IL-12 in response to either opsonized or unopsonized IE was not observed. In contrast, robust secretion of IL-8 was observed, but values exceeded the recommended upper limit of detection of the cytokine bead array assay, and therefore IL-8 response was not further analysed in this study.

### Can IL-1β secretion be induced without IE internalization?

**Figure 5:**
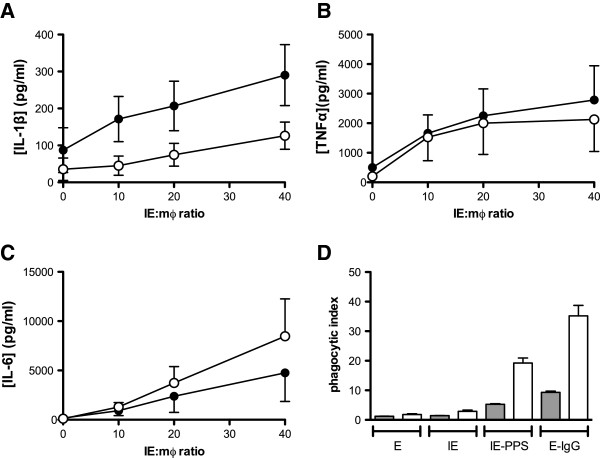
**Fcγ receptor activation by IgG enhances IL-1β but not IL-6 secretion in the absence of IE ingestion.** MDM were grown in Teflon jars for five days, primed for 48 hr with 100 ng/ml IFN-γ and then seeded in triplicate onto wells of 96-well tissue culture plates coated with human IgG (●) or left uncoated (○). After MDM had adhered, they were incubated with unopsonized CS2 IE at an IE:MDM ratio of 20:1. After 24 hr culture medium was collected and analysed for secretion of (**A**) IL-1β (**B**) TNF and (**C**) IL-6. Data represent mean ± SEM of six separate experiments using MDM prepared from independent donor monocytes. Differences at each time point were tested for significance using Wilcoxon matched pairs test; * p<0.05. (**D**) In selected experiments MDM were also seeded in triplicate onto IgG-coated (grey bars) or uncoated (white bars) wells of a separate plate, and phagocytosis of human erythrocytes (**E**), unopsonized CS2 IE, CS2 IE opsonized with pooled immune patient serum (IE-PPS) and human erythrocytes opsonized with rabbit anti-human erythrocyte antibody (E-IgG) was measured. Data represent mean ±SEM of triplicate measurements from a single representative experiment.

Secretion of IL-1β was only observed with opsonized IE. The potential role of Fcγ receptors in this process was therefore considered, in particular whether the ability of these receptors to promote internalization of IE was required or whether signal transduction following binding of opsonized IE to Fcγ receptors was sufficient. To address this question MDM were plated onto tissue culture plates coated with human IgG to trigger Fcγ receptor signalling. MDM were then exposed to unopsonized IE at the indicated IE:MDM ratio and cytokine secretion into the culture medium was quantified. Stimulating Fcγ receptor signalling increased the amount of IL-1β secreted at all concentrations of added IE (Figure 5A). In contrast, there was a decrease in the amount of IL-6 secreted (Figure 5B). Secretion of TNF by MDM plated onto human IgG was not significantly increased under these conditions (Figure 5C). To confirm that IE were not ingested in this system, phagocytosis was measured in parallel cultures (Figure 5D). Under the conditions of these experiments, unopsonized IE were not ingested and internalization of opsonized IE (opsonized either with PPS or with rabbit anti-erythrocyte antiserum) was inhibited, likely due to a decrease in available Fcγ receptors due to their binding to the IgG on the plate surface (Figure 5D). These data indicate that secretion of IL-1β in response to CS2 IE may be stimulated by Fcγ receptor signalling independently of internalization of IE.

### The effect of Fcγ receptor-mediated phagocytosis and signalling on IL-1β processing in response to trophozoite stage CS2-IE

**Figure 6:**
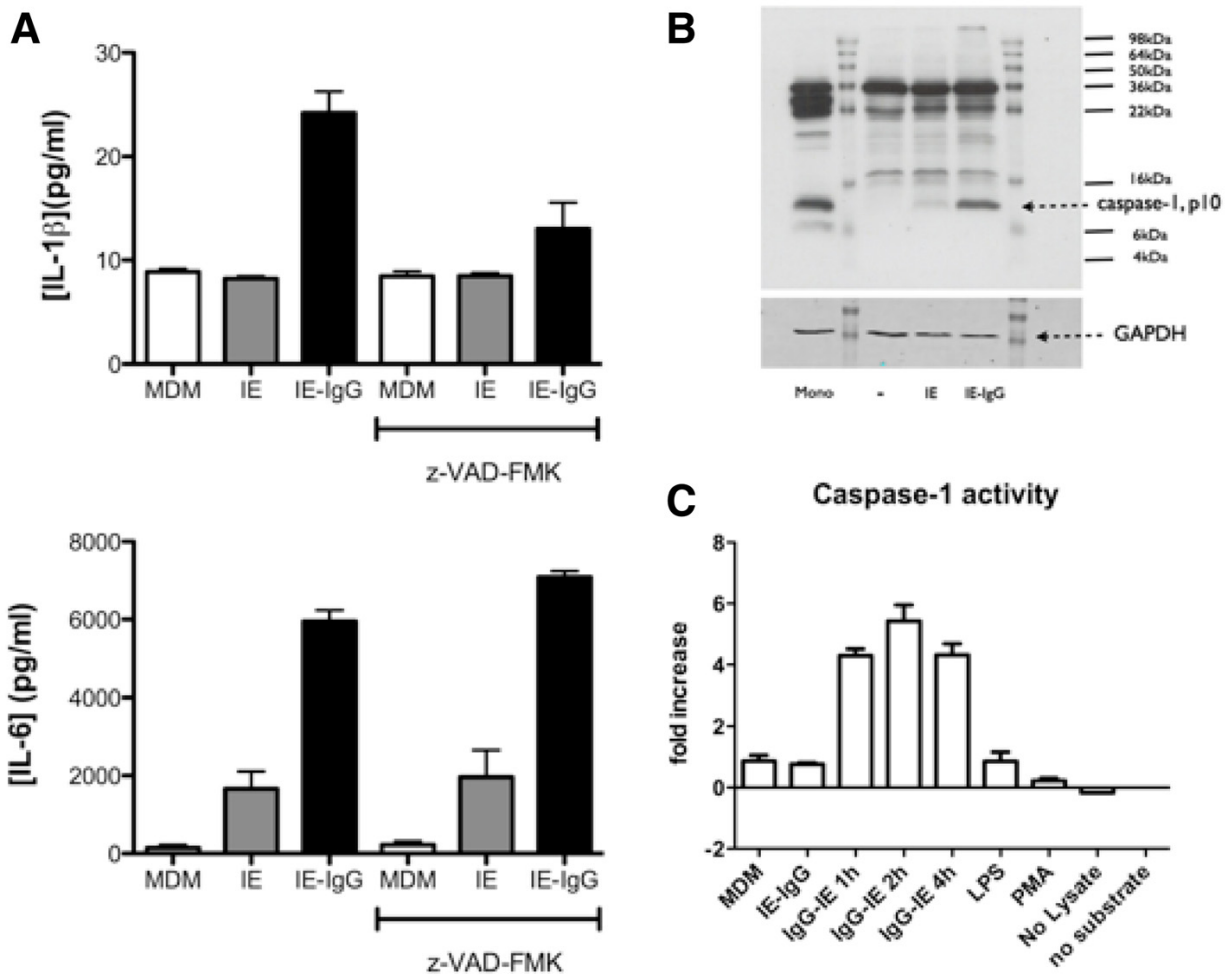
**IL-1β secretion is partially dependent on caspase activity, which is stimulated by IgG opsonization.** (**A**) MDM, cultured in 96-well plates, were primed with 100 ng/ml IFN-γ for 48 hr then pre-incubated as indicated with the pan-caspase inhibitor z-VAD-fmk (10 μM), for 2 hr then exposed to CS2 IE opsonized with rabbit anti-human erythrocyte antibody. Supernatants from replicate wells were pooled and analysed using cytokine bead array. Results represent mean ± SEM from three independent experiments using monocytes from different donors. (**B**) 3 x 10^6^ IFN-γ-primed MDM, cultured in 6 cm dishes, were incubated for 4 hr with 6x10^7^ CS2 IE either unopsonized or opsonized with rabbit anti-human erythrocyte antibody as indicated. Cells were lyzed in RIPA buffer and analysed by immunoblotting using an antibody that recognizes caspase-1 and the 10 kDa cleaved activated subunit of caspase-1. Mono: positive control lysate prepared from autologous purified human monocytes, which show high levels of cleaved caspase-1. MDM incubated in the absence of added trophozoites (−), with unopsonized trophozoites (IE) and with IgG opsonized trophozoites (IE-IgG). Gels were re-probed with anti-GAPDH (lower panel) as a loading control. Results are representative of three experiments using separate donor monocytes. (**C**) MDM cultured and incubated with CS2 IE for the indicated times as in (**B**) were lyzed with a commercial lysis buffer and analysed for caspase-1 activity using a fluorometric assay kit as described in Methods. Data show mean ± SEM fold increase in caspase-1 activity in one experiment, representative of three using separate donor monocytes, each performed in triplicate.

IL-1β secretion requires interleukin 1-converting enzyme (ICE or caspase-1) activation by the inflammasome [42]. Since IL-1β was secreted in response to opsonized but not unopsonized CS2 IE, the question of whether Fcγ receptor phagocytosis and signalling was required for inflammasome activation was addressed. In three independent experiments using monocytes derived from different donors, the effect of caspase inhibition on cytokine secretion in response to both opsonized and unopsonized CS2 IE was measured. In agreement with observations reported above, no IL-1β secretion was observed in response to unopsonized IE, although IL-6 secretion was. Opsonization induced IL-1β secretion and stimulated IL-6 secretion. IL-1β secretion in response to opsonized CS2 IE was inhibited 78% in the presence of 10 mM z-VAD-FMK (Figure 6A) consistent with IL-1β secretion by macrophages being dependent of the inflammasome. As expected, IL-6 secretion was not inhibited by the caspase inhibitor (Figure 6A). MDM were then exposed to opsonized or unopsonized CS2 IE, and cell extracts analysed for activation of caspase-1 by immunoblotting and by enzymatic assay. Exposure to opsonized IE but not unopsonized IE resulted in cleavage of a small proportion of active caspase-1 as evidenced by the accumulation of a 10 kDa cleavage product (Figure 6B). Consistent with this, incubation with opsonized, but not unopsonized, IE caused an increase in caspase-1 proteolytic activity which was maximal 2 hr after exposure to IE (Figure 6C). Thus engagement of Fcγ receptors by immune serum opsonized IE activates components of the inflammasome associated with IL-1β secretion.

## Discussion

The mechanisms of phagocytosis of *P. falciparum* IE are well understood, however the pathways regulating the subsequent inflammatory response, important for control of parasitaemia, are not. Here it is shown that unopsonized CS2 IE, although not internalized by human MDM, stimulate pro-inflammatory cytokine mRNA expression. This is associated with a low level of IL-6 secretion but no IL-1β or TNF secretion. Opsonization by immune serum increases IE internalization via Fcγ receptors without increasing cytokine mRNA levels, and activates components of the inflammasome leading to IL-1β secretion. These data are consistent with recently published studies [35] in which it is shown that HIV infection of MDM inhibits both internalization and cytokine secretion in response to IE-PPS. Internalization of IE is not an absolute requirement for IL-1β secretion since activation of Fcγ receptors via plate-bound IgG also causes IL-1β secretion in response to uningested, unopsonized IE. Thus Fc-receptor stimulation coupled with pattern recognition receptor engagement by IE ligands is sufficient to initiate IL-1β secretion. In contrast, opsonization of IE stimulates IL-6 secretion, likely via a mechanism dependent on ingestion since plate-bound IgG inhibits both ingestion of, and IL-6 secretion in response to, unopsonized IE.

The present study investigated cytokine responses to purified CS2 trophozoite-infected erythrocytes. CS2 is a laboratory-derived *P. falciparum* strain, obtained by selection for CSA binding [36] similar to pregnancy-associated malaria strains. It does not bind the class B scavenger receptor CD36, and consequently CS2-infected erythrocytes are not internalized by macrophages in an unopsonized state. By using this strain, it was possible to dissect the requirement for internalization for some macrophage cytokine responses. The observation that unopsonized IE stimulate NF-κB activation, mRNA expression and some IL-6 secretion was surprising and suggests that surface pattern recognition receptors activate signalling pathways sufficiently to allow for cytokine gene transcription via activation of the NF-kB pathway but that additional signalling pathways must be activated in order to support robust cytokine synthesis and secretion. These additional signalling pathways may be activated following binding to opsonic receptors on the cell surface or to endosomal pattern recognition receptors following phagocytosis.

These observations may suggest that surface pattern recognition receptors, in addition to endosomal receptors, play a role in cytokine elicitation in response to IE. It has been shown that toll-like receptor 2 (TLR2) stimulates pro-inflammatory cytokine production in response to parasite-derived glycosylphosphatidylinositol [43] and TLR4 stimulates TNF secretion in response to peroxiredoxin [44]. TLR9 has been reported to recognize haemozoin [45], possibly complexed with DNA and lipid [46,47]. In contrast, Wu *et al.* demonstrated that cytokine production by murine bone marrow-derived dendritic cells, in response to schizont bursts, were mainly due to recognition by a TLR9-dependent mechanism of protein DNA complexes released from merozoites [48,49] and that haemozoin is inert in this response. Using a mouse model of malaria infection, the same authors showed that the TLR9 response is particularly important in early infection to produce dendritic cell-derived pro-inflammatory cytokines although other mechanisms are required for IL-1β secretion [50]. In the above-mentioned studies, endosomal toll–like receptors were presumed to become activated following delivery of parasite ligands into phagolysosomes. The observations using the CS2 parasite line suggest that activation of pattern recognition receptors and NF-κB signalling occurs in the absence of ingestion. The ligand(s) present on the IE surface which stimulate this response, and the relevant pattern-recognition receptors, remain to be identified. The purified parasite preparations used were essentially without free haemozoin and schizont stages, as determined by microscopy, which is consistent with the observations of poor cytokine responses and low caspase-1 activation with unopsonized IE, since soluble haemozoin stimulates IL-1β release via activation of the NALP3 inflammasome [47]. The lack of IL-1β or TNF secretion in response to unopsonized IE also shows that the experiments on cytokine secretion in response to IE were not confounded by lysis of IE and release of haemozoin from IE during the incubation as this would have induced inflammasome activation and IL-1β secretion [51].

Additional signalling required for robust pro-inflammatory responses may be downstream of Fcγ receptors and/or be activated by internal pattern recognition receptors recognizing ligands in the IE that are released following ingestion of the cells. Whether there is a qualitative difference in the cytokines produced in response to these different pathways remains to be determined. Recent studies have suggested that murine dendritic cells require the presence of multiple Toll-like receptors to induce TNF secretion in response to schizont stage murine malaria parasites since NF-κB nuclear translocation, TNF secretion and dendritic cell activation are abrogated in bone marrow-derived dendritic cells obtained from TLR9, TLR4 and MyD88 knockout mice [52]. These data suggest that schizont ligands require concerted action of several receptors to generate a robust cytokine response, similar to the data presented here, but the role of internalization and of opsonic receptors was not studied.

Data reported herein, using the CS2 parasite strain, differ from those reported using CD36-binding isolates [53]. In the study by Erdman and co-workers, CD36 signalling was shown to inhibit pro-inflammatory cytokine production by macrophages exposed to unopsonized IE. Whether CD36 will also block Fc receptor-induced cytokine production remains to be established. The observation that this does not occur with the CSA-binding strain CS2, has implications for cytokine production in maternal malaria, which involves accumulation of CSA-binding parasites in the placenta. Production of pro-inflammatory cytokines is an important component of the early response against malaria infection but may also contribute to immunopathology. In particular the production of endogenous pyrogens such as TNF, IL-1β and IL-6 following IFN-γ stimulation of monocytes/macrophages may be responsible for fever induction [54] and sustained IL-1β production may be associated with anaemia [55]. The potential roles of pro-inflammatory cytokines in pathology in the setting of maternal malaria are reviewed elsewhere [1]. The ability of opsonized IE to stimulate pro-inflammatory cytokine production by MDM following phagocytosis and its relationship to protection from malaria need to be addressed.

## Conclusions

The data presented in this paper show that generating antibody responses to blood-stage malaria parasites is potentially beneficial both in reducing parasitaemia via Fcγ receptor-dependent macrophage phagocytosis and in generating a robust pro-inflammatory response.

## Abbreviations

CSA: Chondroitin sulphate A; IE: Trophozoite-stage infected erythrocytes; IE-PPS: IE opsonized with pooled immune serum; EMSA: Electrophoretic mobility shift assay; MDM: Monocyte-derived macrophages; IQR: Interquartile range; IRAK4: Interleukin-1 receptor-associated kinase-4; IL-1β: Interleukin-1β; TNF: Tumour necrosis factor (formally TNF-α); IL-6: Interleukin-6.

## Competing interests

The authors declare that they have no competing interests.

## Authors’ contributions

JZ and LEL performed experiments and participated in data analysis. WH performed experiments. SJR designed the study, participated in data analysis and helped to draft the manuscript. AJ designed the study and drafted the manuscript. All authors read and approved the final manuscript.
